# Serological evidence of single and mixed infections of *Rift Valley fever virus*, *Brucella spp*. and *Coxiella burnetii* in dromedary camels in Kenya

**DOI:** 10.1371/journal.pntd.0009275

**Published:** 2021-03-26

**Authors:** Mathew Muturi, James Akoko, Daniel Nthiwa, Bernard Chege, Richard Nyamota, Mathew Mutiiria, Josphat Maina, S. M. Thumbi, Mutono Nyamai, Samuel Kahariri, Rinah Sitawa, Joshua Kimutai, Wilson Kuria, Athman Mwatondo, Bernard Bett

**Affiliations:** 1 Zoonotic Disease Unit Nairobi, Nairobi, Kenya; 2 International Livestock Research Institute, Nairobi, Kenya; 3 Department of Biological Sciences, University of Embu, Embu, Kenya; 4 Center for Epidemiological and Modelling Analysis, University of Nairobi Institute of Tropical and Infectious Diseases, Nairobi, Kenya; 5 Institute of Immunology and Infection Research, University of Edinburgh, Edinburgh, United Kingdom; 6 Paul G Allen School for Global Health, Washington State University, Pullman, Washington, United States of America; 7 Kenya Directorate of Veterinary Services, Ministry of Agriculture, Livestock and Fisheries, Nairobi, Kenya; 8 Food and Agriculture Organization of the United Nations, Nairobi, Kenya; Baylor College of Medicine, UNITED STATES

## Abstract

Camels are increasingly becoming the livestock of choice for pastoralists reeling from effects of climate change in semi-arid and arid parts of Kenya. As the population of camels rises, better understanding of their role in the epidemiology of zoonotic diseases in Kenya is a public health priority. Rift Valley fever (RVF), brucellosis and Q fever are three of the top priority diseases in the country but the involvement of camels in the transmission dynamics of these diseases is poorly understood. We analyzed 120 camel serum samples from northern Kenya to establish seropositivity rates of the three pathogens and to characterize the infecting *Brucella* species using molecular assays. We found seropositivity of 24.2% (95% confidence interval [CI]: 16.5–31.8%) for *Brucella*, 20.8% (95% CI: 13.6–28.1%) and 14.2% (95% CI: 7.9–20.4%) for *Coxiella burnetii* and *Rift valley fever virus* respectively. We found 27.5% (95% CI: 19.5–35.5%) of the animals were seropositive for at least one pathogen and 13.3% (95% CI: 7.2–19.4%) were seropositive for at least two pathogens. *B*. *melitensis* was the only *Brucella* spp. detected. The high sero-positivity rates are indicative of the endemicity of these pathogens among camel populations and the possible role the species has in the epidemiology of zoonotic diseases. Considering the strong association between human infection and contact with livestock for most zoonotic infections in Kenya, there is immediate need to conduct further research to determine the role of camels in transmission of these zoonoses to other livestock species and humans. This information will be useful for designing more effective surveillance systems and intervention measures.

## Author summary

Dromedary camels are well adapted to the arid and semi-arid environment that makes up about 80% of Kenya’s landmass. As such, camels play an important role in the socio-economic wellbeing and food security of pastoralists in the country. However, the species remains relatively neglected in scientific research, one of the main reasons being camels are mostly found in remote, low-income, arid regions of Africa and Asia. We carried out a study to determine the levels of exposure of camels in northern Kenya to *Brucella spp*., *Coxiella burnetii* and *Rift Valley fever virus*, three priority zoonotic pathogens in the country. We found high levels of exposure to the three pathogens, indicating the important role camels might play in the epidemiology of the zoonotic diseases in humans and other livestock. Based on the study findings, we argue for the immediate need for investments in disease surveillance and control strategies for priority zoonotic disease in camels in Kenya.

## Introduction

The emergence of the Middle East respiratory syndrome coronavirus (MERS-CoV) in 2012 and the subsequent detection of the virus in dromedary camels, brought into focus the role of the species as potential reservoirs of endemic and emerging zoonotic diseases in Kenya [1–4]. As the population of camels and their contribution to food security and economy of pastoralists in the country continues to rise, understanding this role is a public health priority [5–7]. The burden and transmission dynamics of zoonotic diseases in Kenyan camels are poorly understood. This has led to the perception of low burden of camel diseases, the consequence of which is continued neglect of the camel industry in resource allocation for disease control and research [8, 9].

Previous studies have identified camels as reservoirs of three of the top priority zoonotic pathogens in Kenya; *Brucella spp*, *Rift Valley fever virus* (*RVFv*) and *Coxiella burnetii*, but data on infection patterns of these pathogens, and potential co-infections in camels is limited [2, 8, 10]. Serological studies, however, show that camels have high seroprevalences and may play an important role in the epidemiology of the three pathogens [2, 11]. Camels have been reported to have the highest *Brucella* and *Coxiella burnetii* infection rates among Kenyan livestock, with one study reporting more than a two-fold increase in *Brucella* prevalence in livestock herds with camels versus those with none [8, 12, 13]. Similarly, camels play an important but underappreciated role in the epidemiology of *RVFV*, one of the pathogens with the highest exposure levels in Kenyan camels [8]. In addition to the high prevalence rates, high camel mortality rates during RVF outbreaks in Kenya and the association of camels with confirmed human RVF cases are indicative of role of camels in the epidemiology of the virus [14–16].

Co-exposure with multiple pathogens has been shown to alter host-pathogen interactions (within and between host pathogen dynamics) which in turn may affect clinical consequences, transmission dynamics and response to prevention and control measures [17–19]. Whereas, advances in diagnostic techniques have increased interest in understanding pathogen interactions, most epidemiological studies in livestock focus on single-pathogen exposures despite the fact that most zoonotic diseases present with similar clinical symptoms and have the same risk factors [20].

Although presence of antibodies for a given pathogen indicates exposure and not necessary active infections, it is an integral component of disease risk assessment. In this study, we investigated the seroprevalences of *RVFV*, *Brucella spp* and *Coxiella burnetii* and characterized *Brucella* species using molecular techniques in camels from northern Kenya counties of Isiolo and Samburu.

## Methods

### Ethics statement

This study did not require ethical approvals from an institutional review board because it was conducted as part of an outbreak investigation by the Kenya Ministry of Agriculture, Livestock and Fisheries and Ministry of Health. However, full approval for the study was given by the Director of Veterinary services in Kenya and local county-level Directors of Veterinary Services in the study area. Although individual informed consent was not required for this investigation, all data were handled as a de-identified set to protect farmers’ privacy and confidentiality.

### Study area

**Fig 1:**
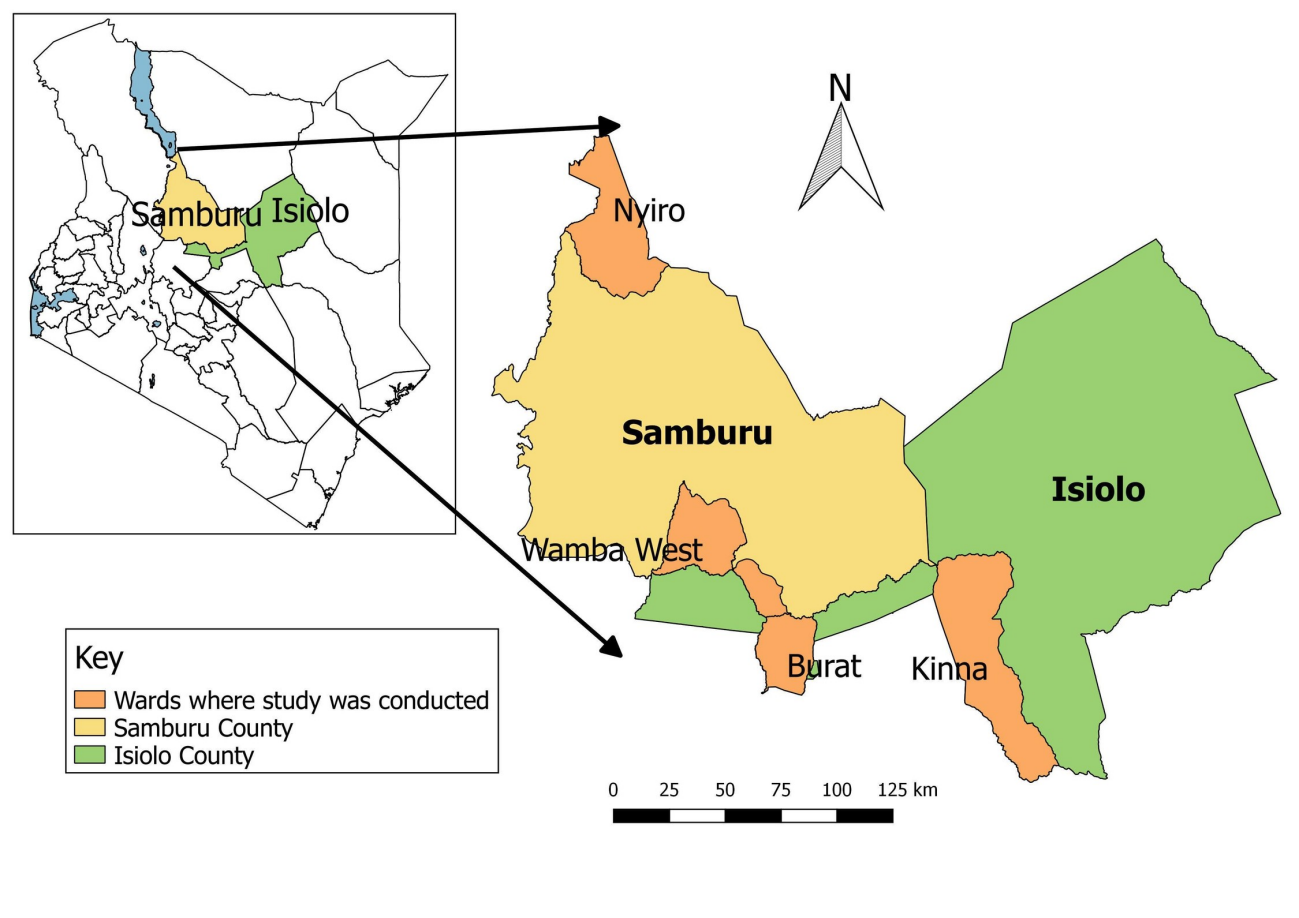
Map of the study area showing the sampled wards (in orange). Shape file used from: https://africaopendata.org/dataset/kenya-counties/shapefile/resource/0b78f25e-494e-4258-96b8-a3ab2b35b121.

We utilized 120 camel sera samples collected from 16 herds during an outbreak investigation of a respiratory syndrome of unknown aetiology in dromedary camels between 22^nd^ and 28^th^ June 2020. Sera samples were collected from herds with history of at-least one camel presenting with a respiratory syndrome in the 3 months preceding the investigation. The investigation was conducted in Isiolo and Samburu counties, both of which are semi-arid, pastoral regions of northern Kenya (Fig 1). In Isiolo, sampling was conducted in Kinna and Burat wards, while in Samburu camel samples were collected from Wamba West and Nyiro wards. Pastoralism, characterized by transhumance movement is the predominant livestock production system in both study areas. Cattle, goats, sheep and camels are the main species kept in the study area, but goats and sheep form the bulk of livestock in the two counties. Isiolo and Samburu are also home to several wildlife conservancies with significant population of free roaming wildlife and human-animal-wildlife interaction.

### Sample collection

Ten millimeters of blood was collected into plain vacutainer tubes from each animal through jugular venipuncture. Serum was then extracted through centrifugation at the laboratories located within county veterinary offices. After serum separation, samples were transferred into 2mls cryovials, labeled and transported in a motorized cool box at 4^o^c to the International Livestock research Institute, Nairobi for laboratory analysis and testing. Study data was collected electronically in Epi-Info (CDC Atlanta, Georgia, USA). Standardized and pre-tested questionnaires were uploaded to smart phones and administered to key informants; primarily camel owners and herders, to collect data on herd and animal level information such as age and sex.

### Laboratory procedure

All serum samples were tested in duplicates for *Brucella*, *RVFV* and *Coxiella burnetii* antibodies using ID screen Brucellosis Serum Indirect ELISA Multispecies kits (IDvet innovative diagnostics, France), ID screen Rift Valley Fever Competition Multispecies ELISA kits (IDvet innovative diagnostics, France) and Q fever (*Coxiella burnetii*) Antibody test kit (IDEXX Laboratories, Westbrook ME, USA) respectively. Samples that tested positive for *Brucella* on ELISA test were further subjected to two PCR assays to detect the genus *Brucella* and a speciation assay to identify *Brucella* species. All the testing was done according to manufacturer standard operating procedures.

Based on the manufacturer’s instruction, the results were interpreted as negative if the calculated S/P% was <30%, positive if the S/P was ≥40% and doubtful if the S/P was in between 30 and 40%.

### Real time PCR detection of *Brucella* spp.

#### DNA extraction from serum samples

Total DNA was extracted from all the 32 Brucella sero-positive samples using QIAamp DNA Blood Mini Kit, while following the manufacturer’s instructions. Briefly, 20 μl of proteinase K solution, 200μl of the serum sample and lysis buffer were added into a sterile 1.5ml Eppendorf tube, vortexed and incubated at 56°C for 10 minutes. Absolute ethanol (200μl) was then added into the mixture and vortexed before applying the lysate onto a QIAamp spin column. The columns were centrifuged at 8000 rpm for one minute followed by a two wash steps using 500μl for each buffer AW1 and AW2 respectively. Centrifugation with buffer AW1 was done at 8000 rpm for 1 minute while AW2 was done at maximum speed for 3 minutes. The DNA was eluted from the spin column using buffer 70μl AE and centrifugation at 8000 rpm for one minute. Eluted DNA was spectrophotometrically checked for quality and quantified using NanoDrop 1000 UV-Vis spectrophotometer (Thermo Scientific, Waltham, MA, USA) and stored at -20°C until when required for analysis.

#### Real-time PCR

Real-time PCR targeting IS711 gene was done to detect the genus *Brucella* in the extracted DNA samples using oligonucleotide primers and probes (S1 Table). The samples were considered PCR-positive for Brucella if they had a genus-specific amplification with a thresh hold value (<40) in one or all the duplicate samples. The genus PCR-positive samples were subsequently subjected to a multiplex speciation assay with *B*. *abortus*, *B*. *melitensis* and *B*. *suis* species-specific oligonucleotide primers and probes. For both genus and species-specific PCR assays, the test samples were considered positive when they had an amplification with cycle threshold (Ct) values <40 for either or both duplicates (S1 Text).

#### Statistical analysis

Field and laboratory data were stored in Microsoft Excel files. They were imported to R statistical computing environment [21] for descriptive and statistical analysis. Variables that were available for analysis included (i) three dependent variables that represented exposure states of the three pathogens: *RVFV*, *Brucella* spp. and *Coxiella burnetii*, recorded as either positive or negative, and two independent factors—age and sex of an animal. Herd and animal identification numbers were used for merging the data.

Seroprevalence for each of the three pathogens and their 95% confidence intervals were estimated using *binom*.*confint* function. An asymptotic method of estimating these intervals was specified for all the tests. Chi-square or Fisher’s exact test (where values were less than 10) were used to assess the significance of statistical associations between independent variables—age and sex and the dependent variables. Additional dependent variables that represented co-occurrence of two or all the target pathogens were also generated and used in similar analyses [22].

Univariable mixed-effect logistic regression models were fitted to data with age and sex being used as predictors in turns. Variables that were statistically significant (P<0.05) in the univariable analyses were then used in mixed effects logistic regression model that had herd as a random effect. Where age and sex were present and significant, their interaction terms were included and tested for their significance using likelihood ratio tests. The Akaike’s Information Criterion (AIC) was used to analyze the goodness of fit of the model. The data is presented in tables and plots. We used a sjstats package to generate Intracluster Correlation Coefficient (ICC), which measures the degree of correlation of prevalence estimates within herds after running the mixed effects model [23]. Intracluster correlation coefficient is a measure of variability of infection between herds.

## Results

We analyzed 120 samples collected from 16 camel herds with no history of vaccination against brucellosis, *Coxiella* and *RVFV*. Majority of the camel samples were from females (72%, n = 86) and camels aged above five years (49%, n = 49) as shown in Table 1.

**Table 1:** Age and sex distribution of sampled camels.

Sex	Age in years	% (n)
**Female**		
	<5	33% (39)
	>5	39% (47)
**Male**		
	<5	27% (32)
	>5	2% (2)

*Brucella* had the highest seropositivity rate of the three pathogens at 24.2% (95% confidence interval [CI]: 16.5–31.8%), followed by *Coxiella burnetii* at 20.8% (95% CI: 13.6–28.1%) and *RVFV* at 14.2% (95% CI: 7.9–20.4%). Co-exposure to two or more pathogens was detected in 15.0% (95% CI: 8.6–21.4%; n = 18) of the samples. Up to 27.5% (95% CI: 19.5–35.5%) of the animals were seropositive for at least one pathogen and 13.3% (95% CI: 7.2–19.4%) were seropositive for at least two pathogens. Of those with co-exposures, 92% (n = 22) had 2 co-exposures detected, the rest of the samples (n = 2) had antibodies to all three pathogens. Co-exposure with Brucella species for both *RVFV* and *Coxiella burnetii* were the most common detections. On multivariate analysis, age was found to be significantly associated with co-exposure to *Brucella* and *Coxiella* as shown in *Table 2*.

**Table 2:** Final multivariable mixed effect model of association between select factors with exposure to one or more pathogens with random effects for herd ID.

Variable and category	Targeted pathogens and their levels of co-exposure among sampled animals											
	*Brucella* spp.		*Coxiella burnetii*		*RVFV*		*Brucella spp*. *+ RVFV*		*Brucella* spp. + *Coxiella burnetii*		*Coxiella burnetii + RVFV*	
	Odds Ratio (95% CI)	P value	Odds Ratio (95% CI)	P value	Odds Ratio (95% CI)	P value	Odds Ratio (95% CI)	P value	Odds Ratio (95% CI)	P value	Odds Ratio (95% CI)	P value
Fixed Effect												
Animal sex												
Male	1 (Ref.)		I (Ref.)		1 (Ref.)		1 (Ref.)		1 (Ref.)		1 (Ref.)	
Female	0.7 (2.6–1.9)	0.499	1.8 (0.6–5.5)	0.278	42.6 (1.0–1789.5)	0.049	1.3 (0.1–22.2)	0.838	7.0 (0.5–93.8)	0.143	2.0 (0.1–29.0)	0.604
Age												
<5 years	1 (Ref.)		1 (Ref.)		1 (Ref.)		1 (Ref.)		1 (Ref.)		1 (Ref.)	
>5 years	1.6 (0.6–4.3)	0.388	1.9 (0.7–4.7)	0.200	13.1 (1.0–174.3)	0.052	7.1 (0.6–77.4)	0.108	9.4 (1.3–69.8)	0.028	10.2 (0.2–457.2)	0.220
Variance associated with random effects	0.77	0.019	0.03	<0.001	14.68	0.005	73.03	0.032	1.67	0.002	5.96	0.034
The intra-herd correlation coefficient (ICC) for herd ID	0.19		0.01		0.82		0.96		0.26		0.64	

### Identification of *Brucella* species in camels

Thirty-two *Brucella* (including three borderline positive samples not included in calculation of seropositivity) sero-positive samples were run through PCR for DNA detection. Half of these samples were positive for *Brucella* on PCR (n = 16). Of these, five samples (32%) were positive for *Brucella melitensis*. Eleven of 16 samples positive for *Brucella* genus on PCR could not be confirmed by the *Brucella* species PCR.

## Discussion

The historical neglect of camels and the perception of the species as hardy and resistant to diseases has contributed to limited understanding of their role in the epidemiology of zoonotic pathogens relative to other livestock [8, 24, 25]. Filling this knowledge gap in Kenya; a country with one of the largest camel population globally, is not only key in the economic development and growth of the camel industry, but in reducing the burden of zoonotic diseases in the country [5].

Brucellosis is one of the most important, yet understudied zoonoses in Kenya [26–28]. Our study found more than one in every five camels had been exposed to *Brucella* spps., which conforms to reported camel brucellosis prevalence range (of up-to 40%) in the East Africa region [26, 29]. Studies conducted in areas of Kenya with similar agro-ecological characteristics and animal husbandry practices in 2012 and 2014, found positivity rates of 11% (ELISA) and 6–18% (serum agglutination test) respectively [30, 31]. The high camel brucellosis prevalence rates in northern Kenya are indicative of the high risk of human infection and interspecies transmission [9, 32]. In literature, camels are reported to be susceptible to *B*. *melitensis* and *B*. *abortus* [33–35]. In our study however, we only detected *B*. *melitensis* after testing for *B*. *abortus*, *B*.*suis* and *B*. *melitensis*. The detection of only *B*. *melitensis* is in agreement with similar reports in literature that surmise that *B*. *melitensis* is more prevalent in African and Middle East camels than *B*. *abortus* [26, 36]. A plausible explanation is that camels are generally herded together with goats as opposed to cattle because of better adaptation of the former to harsher climates; primarily less water requirement, ability to walk longer distances and well adapted dietary preferences [36, 37]. However, our finding should not be deduced to mean that *B*. *melitensis* does not affect Kenyan camels, but rather the need for comprehensive studies on the molecular epidemiology of brucellosis in Kenyan camels [26, 38].

Data on the burden of Q fever in northern Kenyan camels is scarce [8, 39]. However, studies in camels in Central Kenya found high *Coxiella burnetii* positivity rates of between 18.6% - 46%, which is in the range of our 21% prevalence finding [12, 13, 40]. The high *Coxiella* prevalence in camels is a source of public health concern because of the strong association between *Coxiella* camel positivity and human infection [41, 42]. Additionally, the high exposure rates indicate camels might play an important role as reservoirs of *Coxiella* in mixed livestock herds [42–44].

Outbreaks of Rift valley fever have been frequently reported in Kenya since the first detection of the virus in 1931[45, 46]. Traditionally, cattle, sheep and goats are considered to be the species of interest during RVF outbreaks but emerging evidence shows camels play a significant epidemiological role during epizootics [14, 47, 48]. Our study found a positivity rate of 14% which is lower than previously reported rates of 57% and 21% from two studies conducted in 2007 [15, 48]. Other studies in the region have found varying infection rates from 9.6% in Sudan and 45% in Tanzania [49, 50]. The differences in infection rates can be attributed to differences in agro-climatic zones, one of the drivers of RVF infection and different sampling periods; epidemic versus interepidemic period [51, 52]. Our study found a strong association between *Brucella* and *Coxiella* co-exposure exposure and camels above five years. This can be explained by the increased likelihood of exposure to the two pathogens over time due to their endemicity [14, 53]. Intra-herd correlation coefficient values are key in calculating sample sizes for multistage sampling [54]. The ICC for *RVFv* infection was the highest of the three pathogens. This can be inferred to mean *RVFv* infection within a camel herd is highly correlated. This can be explained by the fact that RVF is a vector borne disease that occurs in explosive outbreaks in very specific ecologies, meaning camels in such areas have much higher exposure rates than those that are not [55]. The persistence of *RVFv* antibodies in camels compared to the two other pathogens, a hypothesis supported by the fact that age was significantly associated with *RVFv* exposure in our study, is also a plausible explanation for the high ICC estimate within herds. ICC is an important variable in the calculation of a design effect, a correction factor applied to sample sizes to correct for errors inherent from using cluster sampling, however information on ICCs for most livestock diseases in developing countries are unknown. Given our ICC estimates for exposures and co-exposures with *RVFv* were higher than the 0.2 value assumed for most infectious diseases, there is need for further investigation and to reconsider the outcome for estimation of ICC in camels [56–58].

Co-exposure with multiple pathogens is a common phenomenon in nature [19]. This is especially true for livestock in pastoral communities in Sub-Saharan Africa with no structured disease control programs, a consequence of which is continuous exposure to endemic pathogens [18, 59]. Extrinsic (environmental) and intrinsic (host-specific) factors influence burden and consequence of co-infections, but these are poorly understood for most endemic livestock diseases in sub-Saharan Africa [59, 60]. Although the outcomes of in vivo pathogen interactions are poorly understood, it is known co-infections can result in change in severity of infection, alteration of transmission dynamics and variation in physiologic response which can change effectiveness of diagnostic techniques, control and prevention efforts [18, 53, 61, 62]. Studies on the outcomes of co-infection in livestock provide evidence of antagonistic effects of co-infection on the host. An example is the Th1 and Th2 immunity response to *Toxoplasma gondii* and nematode infection or in *Trypanomosa cruzi* and helminth infections where the former activates Th1 immune response pathways and the latter the Th2 pathways [20, 63]. The three pathogens of interest in the study can affect the immune system; *Brucella* species and *Coxiella burnetii* can infect macrophages while RVFv is can affect monocytes [55, 64–67]. Livestock reared under pastoral production system are highly vulnerable to co-exposure to pathogens due to high population densities, widespread movement and frequent contact from sharing pasture and watering points [42, 68]. Our study evaluated co-exposures with the three pathogens in camels as a start to understanding burden of mixed infection and possible outcomes. This is important because zoonotic spillover pathways and clinical presentation of the three infections are very similar and understanding these co-exposures can inform surveillance strategies and integrated disease control and prevention plans in the long term.

## Conclusion

This study provides evidence of high exposure rates to the three priority zoonotic pathogens in camels in northern Kenya. Considering the increasing value of camels in food nutrition for pastoralists in Kenya, there is need for more research investment to understand the role of camels in the epidemiology of these diseases. Because the endemicity of these diseases is already established, further research should focus on robust studies to estimate the incidence, transmission between livestock species and from animals to humans, and maintenance hosts for these zoonotic infections to guide future control and elimination programs.

## Supporting information

S1 Table Oligonucleotide primers and probes. (DOCX)

S1 Text Supplementary material showing amplification plots for different assays run to identify *Brucella abortus, B. melitensis* and *B. Suis*. (DOCX)

S1 Data Database used in the investigation. (CSV)
