# Supplementary material for: Serological evidence of single and mixed infections of Rift Valley fever virus, Brucella spp. and Coxiella burnetii in dromedary camels in Kenya
Source: PLoS Negl Trop Dis. 2021 Mar 26;15(3):e0009275. doi: 10.1371/journal.pntd.0009275 (PMC7997034; doi:10.1371/journal.pntd.0009275)
Supplement: S1 Table — (DOCX) [file pntd.0009275.s001.docx]

| Target | Gene targeted | Sequences of primers and probes (5’ -3’) | Fluorophore/ quencher | Reference |
| --- | --- | --- | --- | --- |
| *Brucella* spp. | IS711 | Forward: GGC CTA CCG CTG CGA AT  Reverse: TTG CGG ACA GTC ACC ATA ATG  Probe: AAG CCA ACA CCC GGC | FAM/-MGBNFQ | [22] |
| *B. melitensis* | IS711 downstream of BMEI1162 | Forward: AAC AAG CGG CAC CCC TAA AA  Reverse: CAT GCG CTA TGA TCT GGT TAC G  Probe: CAG GAG TGT TTC GGC TCA GAA TAA TCC ACA | Texas Red/BHQ2 | [23] |
| *B.* *abortus* | IS711 downstream of alkB | Forward: GCGGCTTTTCTATCACGGTATTC  Reverse: CATGCGCTATGATCTGGTTACG  Probe: CGCTCATGCTCGCCAGACTTCAATG | JOE/BHQ1 | [23] |
| *B. suis* | 106 bp fragment of BS1330_II0657 locus encoded on chromosome 2 of B. suis | Forward: GCC AAA TAT CCA TGC GGG AAG  Reverse: TGG GCA TTC TCT ACG GTG TG  Probe: TTGCGCTTTTGTGATCTTTGCTTATGG | VIC/MGBNFQ | [23] |

S1 Table. Oligonucleotide primers and probes
