# Supplementary material for: Serological evidence of single and mixed infections of Rift Valley fever virus, Brucella spp. and Coxiella burnetii in dromedary camels in Kenya
Source: PLoS Negl Trop Dis. 2021 Mar 26;15(3):e0009275. doi: 10.1371/journal.pntd.0009275 (PMC7997034; doi:10.1371/journal.pntd.0009275)
Supplement: S1 Text — (DOCX) [file pntd.0009275.s002.docx]

S1 Text.

Supplementary material showing amplification plots for different assays run to identify *Brucella abortus, melitensis* and *Suis.*


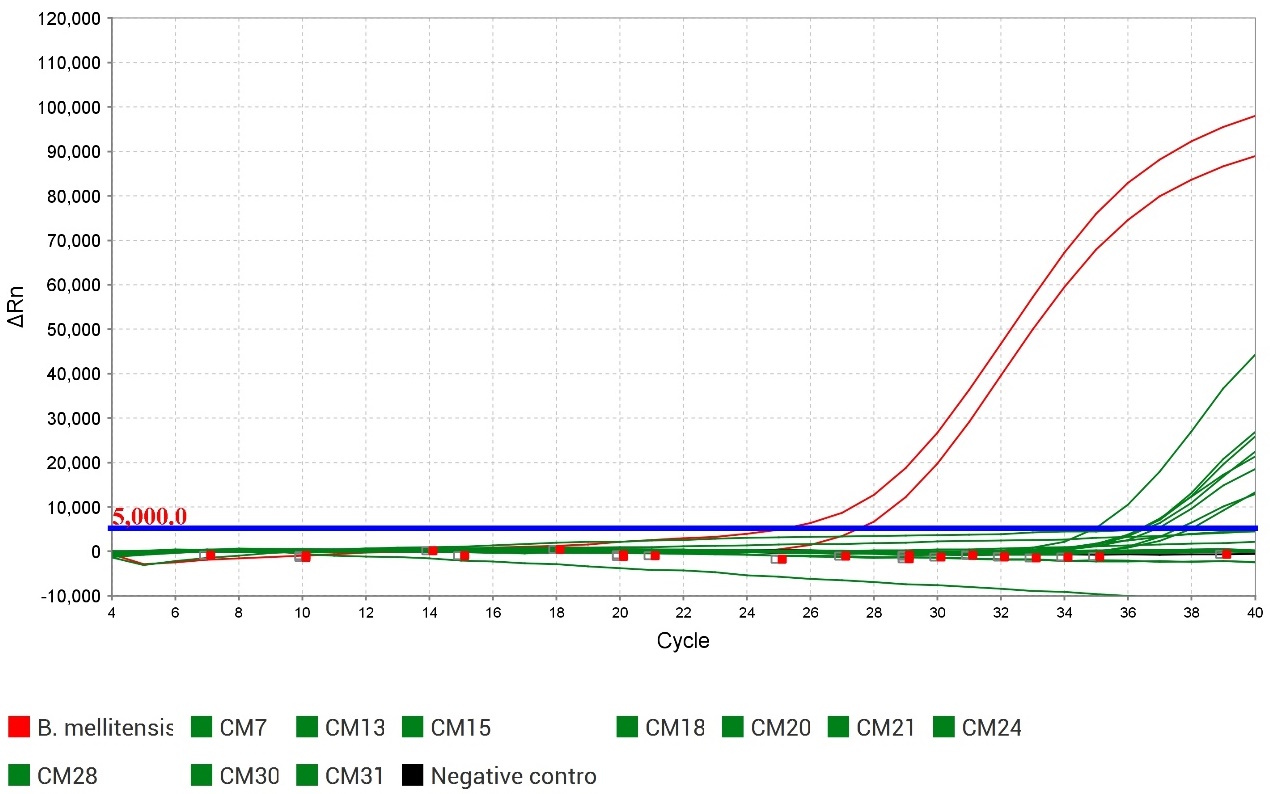


**S1. Figure 1.** The amplification plots showing PCR amplification of *Brucella mellitensis*. The positive control is shown in red colour in the plot while the camel samples and negative controls are shown in green and blue respectively.


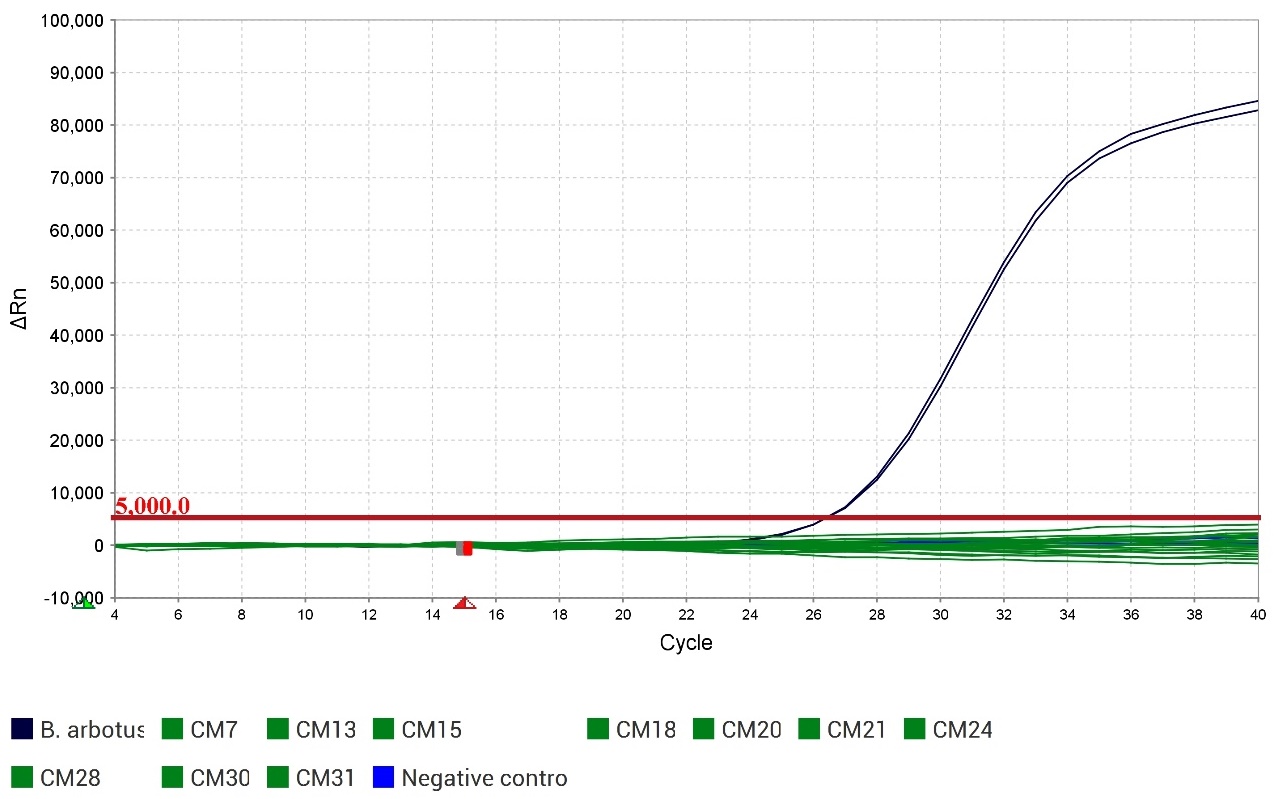


**S1. Figure 2.** The amplification plots showing PCR amplification of Brucella abortus. The positive control is shown in black colour in the plot while the samples and negative controls are shown in green and blue respectively.


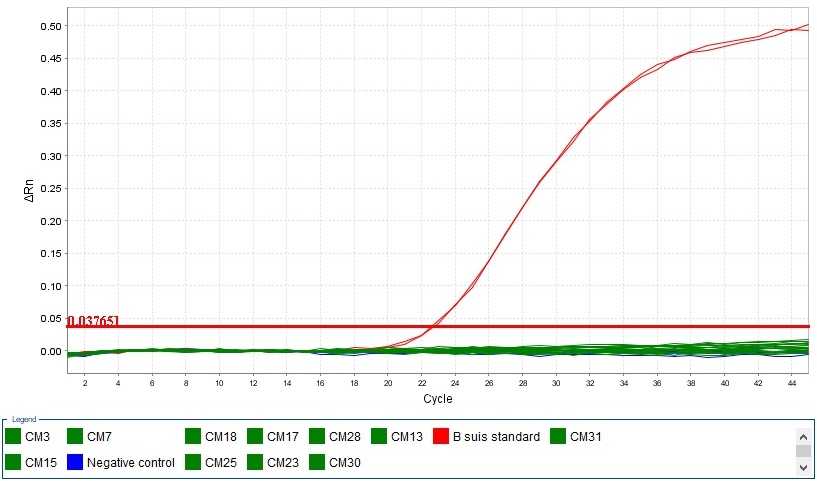


**S1. Figure 3** The amplification plots showing PCR amplification of Brucella suis. The positive control is shown in red colour in the plot while the samples and negative controls are shown in green and blue respectively

**References**

1. Matero P, Hemmilä H, Tomaso H, Piiparinen H, Rantakokko-Jalava K, Nuotio L, et al. Rapid field detection assays for Bacillus anthracis, Brucella spp., Francisella tularensis and Yersinia pestis. Clin Microbiol Infect. 2011. doi:10.1111/j.1469-0691.2010.03178.x
